# The impact of sex and gender on Fibromyalgia Syndrome: data from the Italian Fibromyalgia Registry

**DOI:** 10.1007/s11739-026-04277-2

**Published:** 2026-02-27

**Authors:** Cristina Iannuccelli, Martina Favretti, Giulio Dolcini, Sonia Farah, Marco Di Carlo, Giuseppina Tramontano, Giorgia Ferrari, Fabiola Atzeni, Greta Pellegrino, Piercarlo Sarzi Puttini, Fausto Salaffi, Manuela Di Franco

**Affiliations:** 1 Rheumatology Unit, AOU Policlinico Umberto I–Sapienza University of Rome, Rome, Italy; 2 Department of Molecular Medicine, Sapienza University of Rome, Viale del Policlinico 155, 00161 Rome, Italy; 3 Rheumatology Unit, Department of Molecular and Clinical Sciences, Polytechnic University of Marche, Ancona, Italy; 4 Rheumatology Unit, Department of Medical Specialties, Regional Health Trust 3, Genoa, Italy; 5 Rheumatology Unit, Department of Internal Medicine, University of Messina, Messina, Italy; 6 Rheumatology Unit, IRCCS Ospedale Galeazzi—S. Ambrogio, Milan, Italy; 7 Rheumatology Unit, Department of Clinical and Biomedical Sciences, University of Milan, Milan, Italy; 8 Rheumatology Unit, Department of Medical and Cardiovascular Sciences, Sapienza University of Rome, Rome, Italy

**Keywords:** Fibromyalgia, Sex differences, Gender differences, Disease severity, Physical disability, Differential item functioning (DIF)

## Abstract

Most fibromyalgia (FM) patients are women, with a female-to-male ratio of 3:1. While sex differences in pain perception and description have been reported, the impact of sex and gender on FM severity remains controversial. Additionally, sex-related differential item functioning (DIF) in FM assessment tools has not yet been explored. The primary aim of this study was to analyze sex- and gender-related differences in FM severity using data from a web-based FM registry. The secondary aim was to assess sex-related DIF in three commonly used questionnaires: the Polysymptomatic Distress Scale (PDS), the modified Fibromyalgia Assessment Status (ModFAS), and the revised Fibromyalgia Impact Questionnaire (FIQR). Data from 331 male and 331 female patients, matched for age and body mass index (BMI) and fulfilling ACR 2010/2011 criteria, were retrospectively collected from the Italian Fibromyalgia Registry. Multivariate analyses were conducted on the overall and sex-stratified populations. Sex-related DIF was assessed using a hybrid Ordinal Logistic Regression/Item Response Theory method. Female sex was significantly associated with greater physical impairment, despite no differences in overall disease severity. In stratified analyses, married status influenced disease impact and burden in women, whereas BMI was associated with higher disability in men. Significant sex-related DIF was detected in one item of the Symptom Severity Scale of the PDS. Women with FM experience greater physical impairment than men, despite similar disease severity. Sociodemographic factors influence FM differently across sexes. Despite minor DIF, the three FM-specific questionnaires appear valid for use in both male and female patients.

**Supplementary Information:**

The online version contains supplementary material available at 10.1007/s11739-026-04277-2.

## Introduction

Fibromyalgia (FM) is a leading cause of chronic widespread pain and is characterized by a constellation of symptoms including fatigue, sleep disturbances, cognitive impairment, and mood disorders. Although its aetiology remains unclear, FM is currently regarded as a central sensitization syndrome [1], sharing several features with conditions such as migraine, irritable bowel syndrome, and pelvic pain syndromes [2]. Globally, FM affects 2–3% of the population, with a higher prevalence among women (4.1% vs. 1.4% in men), resulting in a female-to-male ratio of approximately 3:1 [3].

Growing evidence highlights the influence of both sex, the biological differences between males and females, and gender, the socially constructed roles and behaviours associated with being male or female, in shaping the development, clinical expression, and treatment outcomes of FM [4]. Sex differences in the prevalence and clinical manifestations of chronic pain conditions have been documented [5]. Moreover, functional MRI studies have revealed sex-specific patterns of brain activation in response to pain stimuli [6]. Beyond biological factors, psychosocial elements such as coping strategies, gender norms, and societal expectations also significantly influence the chronic pain experience [7–9].

Recognizing these multifaceted influences, current pain management approaches advocate for comprehensive assessments based on the biopsychosocial model, integrating biological, psychological, and social dimensions [10]. Patient-reported outcomes (PROs) are now considered essential tools to evaluate symptoms, functionality, and quality of life from the patient’s perspective [11]. However, sex- and gender-related differences in symptom perception may introduce biases in PROs, potentially limiting the comparability of outcomes across these groups. Differential item functioning (DIF) analysis offers a methodological approach to identify and adjust for such biases, ensuring fair and accurate measurement [12].

Despite growing evidence of sex and gender influences in chronic pain, their specific impact on FM symptomatology and disease severity remains debated [13]. Furthermore, the potential existence of sex- and gender-related biases in commonly used FM severity measures has not been fully explored. In this study, we aimed to investigate sex- and gender-related differences in symptom burden and disease severity in FM, using data obtained retrospectively from a national registry. A further objective was to examine whether sociodemographic factors exert different effects on disease impact and severity across sexes. Additionally, we aimed to assess the presence of sex-related differential item functioning (DIF) in three FM-specific questionnaires.

## Methods

### Subjects

The study population included female and male patients, diagnosed with FM according to the 2010/2011 American College of Rheumatology (ACR) criteria [14]. Participants were recruited from five Italian rheumatology centers. In the multicenter population patients were classified based on their biological sex (male vs. female), and we are not aware of any patient reporting identifying with a gender other than their biological or as non-binary. A one-to-one matching procedure was applied, pairing each male patient with a female counterpart matched for age and body mass index (BMI). Diagnosis was confirmed by rheumatologists with at least 10 years of experience, following a comprehensive physical examination and laboratory testing as per the European League Against Rheumatism (EULAR) guidelines [15]. Exclusion criteria included conditions potentially confounding FM assessment, such as cardiovascular diseases, moderate-to-severe pulmonary disorders, thyroid abnormalities, orthopedic restrictions, inflammatory rheumatic diseases, and psychiatric conditions.

The study protocol was approved by the Ethics Committee of the Università Politecnica delle Marche (Comitato Unico Regionale—ASUR Marche, No. 1970/AV2) and by the review boards of all participating centers. All patients provided written informed consent prior to participation.

### Measurement

Data were retrospectively collected from the Italian Fibromyalgia Registry, initially developed to define disease severity thresholds [16]. Recorded variables included sociodemographic characteristics (sex, age, marital status, education level, BMI) and disease-specific measures: the Polysymptomatic Distress Scale (PDS), the Modified Fibromyalgia Assessment Status (ModFAS), and the Revised Fibromyalgia Impact Questionnaire (FIQR).

#### Polysymptomatic Distress Scale (PDS)

The PDS is calculated by summing the Widespread Pain Index (WPI, 0–19) and the Symptom Severity Scale (SSS, 0–12), with a total score ranging from 0 to 3; higher scores indicate more severe disease [14].

#### Modified Fibromyalgia Assessment Status (ModFAS)

The ModFAS assesses fatigue and sleep quality (two 0–10 Numeric Rating Scales, NRS) and the extent of pain in 19 body areas. The final score (0–39) is the sum of the two NRS scores and the body map score [17].

#### Revised Fibromyalgia Impact Questionnaire (FIQR)

The FIQR evaluates the previous week's impact of FM across 21 items divided into physical function (9 items), symptoms (10 items), and overall impact (2 items). Scores are combined to yield a total score ranging from 0 to 100, with higher scores indicating greater severity [18].

### Statistical analysis

Descriptive statistics were performed, with categorical variables expressed as frequencies and percentages, and continuous variables as medians and interquartile ranges (IQR), due to the non-normal distribution as verified by the Shapiro–Wilk test. Group comparisons for continuous variables were conducted using the paired signed-rank test, while chi-squared tests were used for categorical variables. To control the false discovery rate (FDR) due to multiple comparisons, p-values were adjusted using the Benjamini–Hochberg procedure [19].

Multiple linear regression models were built to examine the impact of sex on disease severity, with the total scores of PDS, ModFAS, and FIQR (and their subscales) as dependent variables. Sex was the main independent variable, and age, BMI, education, and marital status were included as covariates. Further, analyses were stratified by sex to assess differential effects on disease severity and impact. The Benjamini–Hochberg method was applied to adjust for multiple comparisons [19].

Before performing differential item functioning (DIF) analyses, dimensionality was assessed. Dimensionality of the FIQR was addressed based on prior validation studies demonstrating its multidimensional structure [18]. Accordingly, it is considered methodologically appropriate to compute and interpret not only the total score but also the three domain scores (physical function, symptoms, and overall impact) separately [20]. Since the physical function and symptoms domains have been shown to be unidimensional [18], DIF analysis was restricted to these two subscales. For PDS and ModFAS, unidimensionality was tested via confirmatory factor analysis (CFA), considering Comparative Fit Index (CFI) and Tucker-Lewis Index (TLI) ≥ 0.95 and a Root Mean Square Error of Approximation (RMSEA) ≤ 0.06 as indicators of acceptable fit [21].

DIF was assessed using a hybrid Ordinal Logistic Regression/Item Response Theory (OLR/IRT) approach [22], capable of detecting both uniform DIF (consistent across the trait level) and non-uniform DIF (trait-dependent). For each item, three ordinal logistic regression models were fitted: the first included only the total score of the scale, the second added sex as a group variable, and the third incorporated an interaction term between sex and total score. Overall DIF was assessed by comparing the baseline and interaction models through a likelihood ratio test (LRT). Uniform DIF was detected by comparing the baseline and group models, while non-uniform DIF was tested by comparing the group and interaction models. Bonferroni correction was applied to adjust for multiple testing by dividing the alpha level (0.05) by the number of items in each scale on which the DIF analysis was performed [23]. To ensure clinical relevance and avoid detecting trivial effects, DIF was considered substantial only when the difference in pseudo-R^2^ between models was at least 2% [24].

All analyses were performed using R software (version 4.4.0).

## Results

### Descriptive statistics and preliminary analysis

The study included 331 male and 331 female FM patients, matched for age and BMI. Most participants were married (41.9% males, 64% females) and had completed high school (52.2% in both groups) (Table 1).

**Table 1:** Demographic characteristics among FM patients

	Female (n = 331)		Male (n = 331)
Age (year), median; IQR	56; 48–62	53; 45–62	
BMI^a^, median; IQR	24,1; 21.5–28.124,1; 21.5–28.1	25; 22.9–27.6	
Education, n (%)			
Primary/Secondary school	89 (26.8)	37 (11.2)	
High school	173 (52.2)	173 (52.2)	
University	69 (20.8)	121 (36.5)	
Marital status, n (%)			
Single	30 (9.0)	85 (25.7)	
Married	212 (64.0)	139 (41.9)	
Divorced/Separated	79 (23.8)	102 (30.8)	
Widowed	10 (3.0)	5 (1.5)	

^a^Body Mass Index (BMI)

Univariate analysis showed females had higher disease severity across all measures (Table 2). Median PDS (21 [16–25] vs. 18 [12–24], p < 0.001), ModFAS (28 [22–32] vs. 25 [18–31], p < 0.001), and FIQR scores (65 [45–78.5] vs. 54 [37.5–74], p < 0.001) were significantly higher in females. Subscale analyses confirmed greater disease burden in females.

**Table 2:** Comparison of total and subscale scores of PDS, ModFAS, and FIQR in male and female FM patients

	Female (median; IQR)	Male (median; IQR)	p value^a^
PDS^b^ total score	21; 16–25	18; 12–24	< 0.001
WPI^c^ domain score	11; 8–14	10; 7–14	0.04
SSS^d^ domain score	9; 7–11	8; 6–10	< 0.001
ModFAS^e^ total score	28; 22–32	25; 18–31	< 0.001
ModFAS-WPI domain score	11; 8–14	10; 7–14	0.04
ModFAS-sleep domain score	6; 4–8	6; 2–7.5	< 0.001
ModFAS-fatigue domain score	9; 7–10	7; 5–9	< 0.001
FIQR^f^ total score	65; 45–78.5	54; 37.5–74	< 0.001
FIQR physical function domain	18; 11–23	14; 7–21	< 0.001
FIQR overall impact domain	12; 7.5–17	11; 6–16	0.04
FIQR symptoms domain	34; 26–41	31; 22.5–39	< 0.001

^a^Adjusted for Benjamini–Hochberg correction

^b^Polysymptomatic Distress Scale (PDS)

^c^Widespread Pain Index (WPI)

^d^Symptoms Severity Scale (SSS)

^e^Modified Fibromyalgia Assessment Status (ModFAS)

^f^Revised Fibromyalgia Impact Questionnaire (FIQR

Pain distribution differed between sexes: neck pain was most common in both groups but more prevalent in females (81% vs. 72%, p = 0.01). Males more often reported chest (48% vs. 38%, p = 0.02) and left upper arm pain (60% vs. 52%, p = 0.04). Females reported higher rates of pain in the right shoulder (76% vs. 67%, p < 0.001), hips, right lower leg, lower back, and abdomen (all p < 0.02).

Regarding the SSS subscale, females reported greater fatigue (SSS-1), sleep disturbance (SSS-2), and cognitive symptoms (SSS-3) (all p < 0.001). They also more frequently reported lower abdominal cramps (64% vs. 54%, p = 0.02) and depression (75% vs. 67%, p = 0.02) (Supplementary Table S1).

Similarly, in the FIQR item analysis, females scored higher in nearly all items, particularly for fatigue severity (FIQR-13). Males showed higher scores for feeling overwhelmed (FIQR-11) and pain severity (FIQR-12), although females still had overall higher values (Supplementary Table S2). No differences emerged for daily life impact, stiffness, depression, or balance problems.

### Multiple linear regression analysis

Regression models for the PDS and ModFAS scores identified a positive association only with the FIQR total score (β = 0.225 and β = 0.296, both p < 0.001), and a negative association with age (β = − 0.036, p = 0.04; β = − 0.031, p = 0.01). No direct association with female sex was found, except for the SSS domain score (β = 0.737, p < 0.001) (Table 3).

**Table 3:** Multiple linear regression analysis exploring associations between clinical/sociodemographic factors and disease severity scores (PDS, ModFAS, WPI, SSS)

Independent variables	Model 1 (PDS^d^ total) β (p value^a^)	95% CI model1 (lower; upper)	Model 2 (WPI^e^) β (p value^a^)	95% CI model2(lower; upper)	Model 3 (SSS^f^) β (p value^a^)	95% CI model3 (lower; upper)	Model 4 (ModFAS^g^ total) β (p value^a^)	95% CI model4 (lower; upper)
Female sex	0.304 (0.5)	− 0.387; 0.995	− 0.390 (0.3)	− 0.975; 0.194	**0.737 (< 0.001)**	0.415; 1.060	0.123 (0.8)	− 0.639; 0.886
Age	**− 0.036 (0.04)**	− 0.065; − 0.006	− 0.014 (0.5)	− 0.039; 0.010	**− 0.031 (0.01)**	− 0.067; 0.004	**− 0.014 (0.04)**	− 0.075; − 0.010
BMI^b^	− 0.036 (0.4)	− 0.113; 0.039	− 0.009 (0.7)	− 0.074; 0.054	− 0.021 (0.1)	− 0.035; − 0.007	− 0.042 (0.7)	− 0.101; 0.073
Education								
High school	− 0.297 (0.9)	− 1.056; 0.716	− 0.120 (0.9)	− 0.872; 0.631	− 0.201 (0.9)	− 0.615; 0.212	− 1.142 (0.1)	− 2.103; − 0.180
University	− 0.164 (0.8)	− 1.060; 0.969	− 0.204 (0.8)	− 1.062; 0.654	− 0.025 (0.9)	− 0.499; 0.447	− 0.806 (0.4)	− 1.877; 0.264
Marital status								
Married	0.909 (0.09)	− 0.439; 1.435	0.763 (0.09)	− 0.064; 1.592	0.167 (0.4)	− 0.288; 0.624`	0.890 (0.1)`	− 0.328; 2.109
Divorced	0.471 (0.8)	− 0.677; 1.333	0.063 (0.8)	− 0.789; 0.915	0.369 (0.8)	− 0.100; 0.840	0.360 (0.8)	− 0.813; 1.534
Widowed	1.154 (0.8)	− 1.526; 2.828	0.840 (0.8)	− 1.170; 2.851	0.259 (0.8)	− 0.848; 1.367	2.017 (0.8)	0.031; 4.003
FIQR^c^ total	**0.225 (< 0.001)**	0.210; 0.240	**0.130 (< 0.001)**	0.117; 0.142	**0.095 (< 0.001)**	0.088; 0.102	**0.296 (< 0.001)**	0.280; 0.312

Bold denotes the presence of statistically significant associations, with* p*-value 0.05

^a^Adjusted for multiple comparisons (Benjamini–Hochberg correction)

^b^Body Mass Index (BMI)

^c^Revised Fibromyalgia Impact Questionnaire (FIQR)

^d^Polysymptomatic Distress Scale (PDS)

^e^Widespread Pain Index (WPI)

^f^Symptoms Severity Scale (SSS)

^g^Modified Fibromyalgia Assessment Status (ModFAS)

**Fig. 1:**
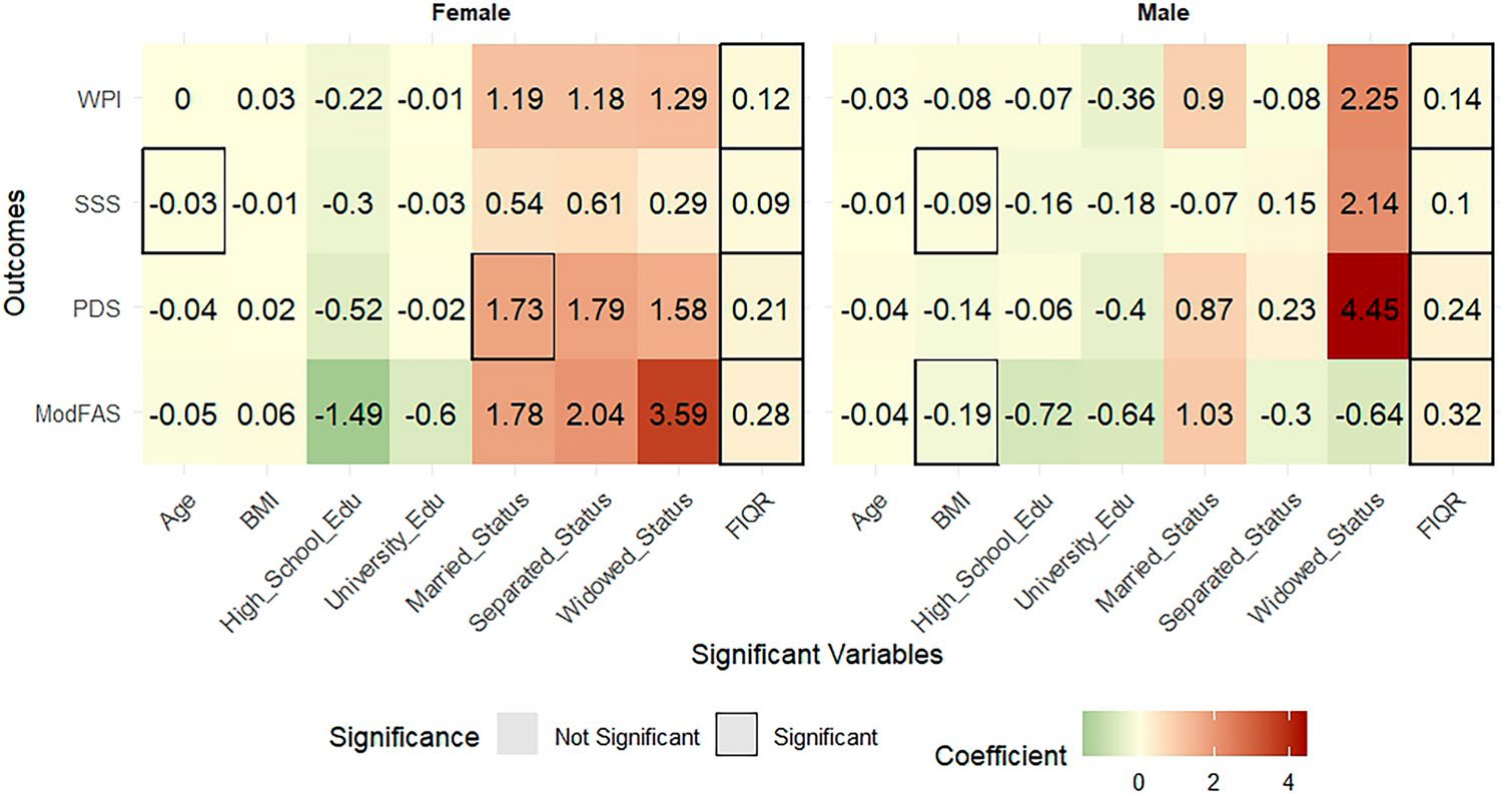
Multiple linear regression analysis stratified by sex: associations between clinical/sociodemographic factors and PDS, ModFAS, WPI, and SSS scores. Significant associations are outlined in black

Stratified analysis revealed that, in females, married status was positively associated with PDS (β = 1.730, p = 0.04) and age negatively with SSS (β = − 0.034, p < 0.001). In males, BMI was negatively associated with ModFAS and SSS scores (β = − 0.190, p = 0.02; β = − 0.092, p = 0.02). In both sexes, FIQR scores remained positively associated with predictors (Fig. 1).

Regression analyses using FIQR total scores showed positive associations with PDS (β = 2.521, p < 0.001) and BMI (β = 0.529, p < 0.001), and a negative association with married status (β = − 5.413, p = 0.009). Similar trends were found for FIQR domains: physical function, overall impact, and symptoms. Only the physical function domain showed a significant positive association with female sex (β = 1.686, p = 0.001) (Table 4).

**Table 4:** Multiple linear regression analysis of associations between clinical/sociodemographic factors and FIQR total score and its domains (Physical Function, Overall Impact, and Symptoms)

Independent variables	Model 5 (FIQR^d^ total) β (p value^a^)	95% CI model5 (lower; upper)	Model 6 (FIQR physical function) β (p value^a^)	95% CI model6 (lower; upper)	Model 7 (FIQR overall impact) β (p value^a^)	95% CI model7 (lower; upper)	Model 8 (FIQR symptoms) β (p value^a^)	95% CI model8 (lower; upper)
Female sex	2.656 (0.06)	0.304; 5.007	**1.686 (0.001)**	0.760; 2.613	− 0.002 (0.9)	− 0.764; 0.758	0.957 (0.2)	− 0.182; 2.098
Age	− 0.009 (0.8)	− 0.103; 0.084	0.014 (0.6)	− 0.024; 0.052	− 0.008 (0.6)	− 0.041; 0.023	− 0.022 (0.5)	− 0.068; 0.023
BMI^b^	**0.529 (< 0.001)**	0.277; 0.781	**0.184 (0.001)**	0.087; 0.280	**0.114 (0.01)**	0.030; 0.197	**0.265 (< 0.001)**	0.139; 0.391
Education								
High school	− 0.096 (0.9)	− 3.017; 2.823	− 0.120 (0.9)	− 1.287; 1.041	0.066 (0.9)	− 0.917; 1.051	0.011 (0.9)	− 1.490; 1.513
University	− 3.395 (0.2)	− 6.533; − 0.257	− 0.879 (0.4)	− 2.159; 0.401	− 1.470 (0.08)	− 2.589; − 0.351	− 0.880 (0.4)	− 2.537; 0.776
Marital status								
Married	**− 5.413 (0.009)**	− 8.704; − 2.123	**− 1.596 (0.03)**	− 2.910; − 0.283	**− 1.470 (0.01)**	− 2.657; − 0.507	**− 2.180 (0.02)**	− 3.700; − 0.660
Divorced	− 0.510 (0.8)	− 3.731; 2.710	0.203 (0.8)	− 1.119; 1.526	− 1.582 (0.8)	− 1.306; 0.918	− 0.234 (0.8)	− 1.777; 1.307
Widowed	− 0.831 (0.9)	− 8.765; 7.101	1.045 (0.8)	− 2.526; 4.616	− 0.070 (0.9)	− 2.855; 2.713	− 1.417 (0.8)	− 4.959; 2.124
PDS^c^ total	**2.521 (< 0.001)**	2.356; 2.686	**0.790 (< 0.001)**	0.716; 0.843	**0.531 (< 0.001)**	0.475; 0.586	**1.179 (< 0.001)**	1.088; 1.269

Bold denotes the presence of statistically significant associations, with* p*-value 0.05

^a^Adjusted for multiple comparisons (Benjamini–Hochberg correction)

^b^Body Mass Index (BMI)

^c^Polysymptomatic Distress Scale (PDS)

^d^Revised Fibromyalgia Impact Questionnaire (FIQR)

**Fig. 2:**
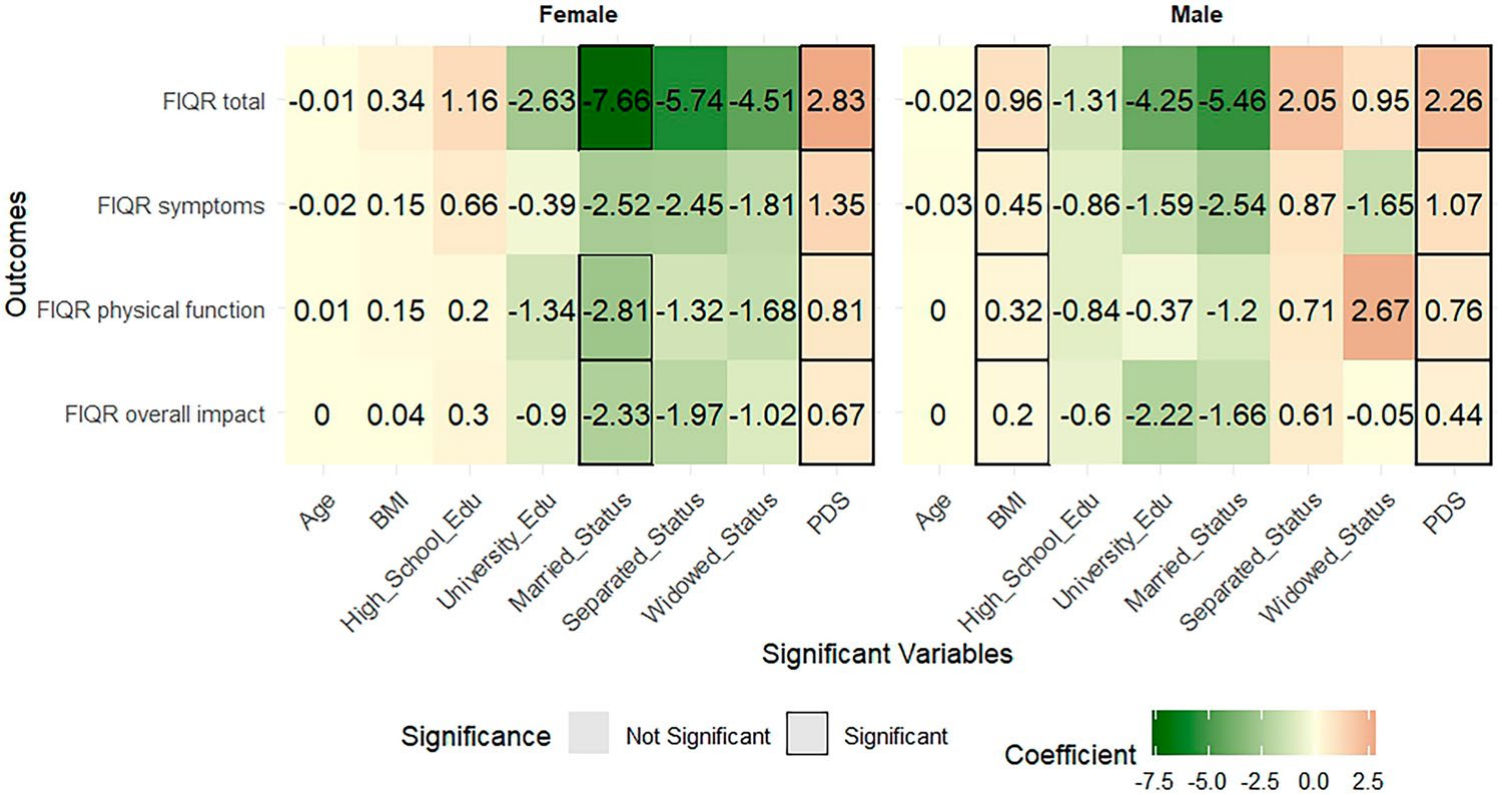
Multiple linear regression analysis stratified by sex: associations between clinical/sociodemographic factors and FIQR total score and its domains. Significant associations are outlined in black

Sex-stratified regression indicated that, in females, married status was negatively associated with FIQR total and domain scores (all p < 0.02). In males, BMI was positively associated with all FIQR domains (p < 0.05). Across both groups, all predictors were positively associated with the PDS (Fig. 2).

### Dimensionality

CFA results confirmed the multidimensionality of the PDS (CFI = 0.533; RMSEA = 0.120) and ModFAS (CFI = 0.489; RMSEA = 0.141). Among the subscales, only the SSS demonstrated unidimensionality (CFI = 0.977; RMSEA = 0.06). Consequently, DIF analysis focused on the SSS subscale.

### Differential item functioning

DIF analysis identified a relevant non-uniform DIF for the SSS-4 item (headache) (χ^2^ = 26.2, p < 0.001, pseudo-R^2^ = 0.0335).

In the FIQR, uniform DIF was detected in FIQR-4 and FIQR-5 (physical function domain) and FIQR-13 and FIQR-15 (symptoms domain). Non-uniform DIF was observed for FIQR-16 (depression severity). However, no items showed meaningful DIF based on the pseudo-R^2^ threshold of ≥ 2% (Supplementary Table S3).

## Discussion

This real-world study, conducted on a large cohort of FM patients, identified sex-related differences in disease impact and functional disability, underscoring the complex interplay of biological and sociocultural factors in shaping the overall disease burden. Data were obtained from the Italian Fibromyalgia Registry and included patients recruited at five centres distributed across northern, central, and southern Italy, thereby accounting for potential regional variations [25].

Previous studies examining the influence of sex and gender on FM severity and impact have produced conflicting results. Some reported greater pain intensity and symptom severity in women [26–28], along with higher total FIQR scores [29, 30], and more pronounced impairment in physical functioning [29] and symptom burden [30]. In contrast, other studies found no significant sex-related differences in symptoms severity [29, 30] or overall disease burden [26, 31]. Conversely, some investigations reported greater disability and disease burden in men [32, 33]. However, these last studies relied on the 1990 ACR criteria [34], which may have underestimated FM prevalence in men and selectively identified more severe cases [35], thus limiting comparability. Importantly, many of these findings are weakened by the small number of male participants and the lack of adjustment for potential confounders. Moreover, it has been hypothesized [29, 30] that psychometric limitations of the FIQR could hinder its ability to capture sex and gender differences. In our analysis, however, differential item functioning across FIQR items was minimal, suggesting that the instrument is free of systematic interpretation bias and is appropriate for cross-sex comparisons of FM impact.

After adjusting for potential confounders, our results showed no significant sex differences in psychological distress or overall disease severity. With respect to disease impact, women tended to report higher FIQR scores than men, although these differences did not reach statistical significance. Notably, analysis of the FIQR physical function domain revealed significantly greater functional impairment in women, even in the absence of differences in overall disease severity. This finding is consistent with the observations of Rutledge et al. [36], who identified male sex as a predictor of better physical functioning.

Evidence suggests [37] that sex-related differences in disability due to chronic pain may arise not only from biological factors but also from psychosocial influences, including emotional responses, cognitive patterns, previous experiences, and cultural norms. Several studies [38–40] have further highlighted the role of sociocultural factors in shaping FM severity and its associated burden. However, the interaction between sex and these factors remains underexplored, with the exception of Atzeni et al. [31], who reported higher disease impact among separated or divorced men.

In our cohort, marital status emerged as a significant determinant of disease burden. Married patients reported lower disease impact and functional disability compared to unmarried patients. Stratified analyses confirmed this association particularly in women, among whom being married was linked to better physical functioning and reduced overall disease impact. These findings suggest that family and emotional context may have a more favourable influence on women’s disease experience, likely due to greater psychological support, the availability of a caregiver, and more advantageous social conditions. This aligns with prior research demonstrating that daily partner interactions affect coping strategies in chronic pain patients [41], with effects varying by the sex of both patient and partner [42]. For instance, happily partnered women report lower pain catastrophizing and better coping, resulting in reduced disability compared to unpartnered or unhappily partnered women [43].

Obesity has also been associated with increased disability and symptom burden in FM [38]. Although in our cohort the median BMI fell within the normal-to-mildly overweight range, higher BMI was nevertheless a predictor of greater disability and disease burden. Stratified analysis revealed that this association was significant particularly among men. This pattern may reflect sex-specific differences in fat distribution: men predominantly accumulate visceral fat, whereas women tend to store subcutaneous fat [44]. Visceral fat has been linked to increased disability risk in men [45, 46], likely due to its association with metabolic dysfunction and decline in muscle strength. Taken together, these findings suggest that body composition represents an additional biological factor that may interact with sex to influence the clinical burden of FM, further reinforcing the importance of a multidimensional approach when evaluating disease impact.

This study has some limitations. Firstly, its cross-sectional design precludes conclusions regarding causality or the temporal evolution of sex differences in FM. Longitudinal studies are necessary to address this limitation. Secondly, our sample was drawn from tertiary care clinics, and therefore the findings may not be generalizable to the broader FM population. Finally, given the multidimensionality of the questionnaires used, DIF analysis was limited to the unidimensional subscales, which may only partially represent the broader constructs assessed.

## Conclusion

In conclusion, this study confirms the complexity of FM, underscoring the role of both biological and sociocultural factors as key determinants of overall disease impact. It also highlights the existence of sex-related differences in how the disease affects patients’ activity of daily living. Moreover, our findings illustrate a complex interplay between sociodemographic variables, such as BMI and marital status, and clinical outcomes, emphasizing that these associations may differ across sexes. To the best of our knowledge, this is the first study to include such a large number of male patients and to assess the effect of sex while adjusting for potential confounders. In addition, it is the first to apply DIF analysis to widely used FM assessment tools, demonstrating the absence of systematic bias in the FIQR physical function and symptom domains, while identifying non-uniform DIF in one SSS item. Future research is warranted to replicate these findings and to further explore the mechanisms underlying sex-related differences in FM, while also addressing the limitations of the present study.

## Supplementary Information

Below is the link to the electronic supplementary material.

Supplementary file1 (PDF 180 KB)

## Data Availability

The data underlying this article will be shared on reasonable request to the corresponding author.
