# Supplementary material for: The impact of sex and gender on Fibromyalgia Syndrome: data from the Italian Fibromyalgia Registry
Source: Intern Emerg Med. 2026 Feb 27;21(4):1193–202. doi: 10.1007/s11739-026-04277-2 (PMC13263255; doi:10.1007/s11739-026-04277-2)
Supplement: Supplementary file 1 — Supplementary file1 (PDF 180 KB) [file 11739_2026_4277_MOESM1_ESM.pdf]

Internal and Emergency Medicine (Gender Medicine)

The impact of sex and gender on Fibromyalgia Syndrome: data from the Italian Fibromyalgia Registry

Martina Favretti, Cristina Iannuccelli, Giulio Dolcini, Sonia Farah, Marco Di Carlo, Giuseppina Tramontano, Giorgia Ferrari, Fabiola Atzeni, Greta Pellegrino, Piercarlo Sarzi Puttini, Fausto Salaffi, Manuela Di Franco.

Corresponding author: Giulio Dolcini, Department of Molecular Medicine, Sapienza University of Rome, Rome, Italy. Email: giulio.dolcini@uniroma1.it

Supplementary Table S1. WPI and SSS single item frequencies and scores among females and males affected by FM

	Female	Male	*p-value**
WPI[a] 1 *jaw, left* (n yes; %)	120; 36	131; 39	ns
WPI2 *shoulder grindle, left* (n yes; %)	250; 75	229; 69	ns
WPI3 *upper arm, left* (n yes; %)	173; 52	201; 60	0.04
WPI4 *lower arm, left* (n yes; %)	162; 48	187; 56	ns
WPI5 *jaw, right* (n yes; %)	120; 36	137; 41	ns
WPI6 *shoulder grindle, right* (n yes; %)	253; 76	222; 67	< 0.001
WPI7 *upper arm, right* (n yes; %)	187; 56	166; 50	ns
WPI8 *lower arm, right* (n yes; %)	164; 49	163; 49	ns
WPI9 *hip (buttock, trochanter), left* (n yes; %)	204; 61	169; 51	0.01
WPI10 *upper leg, left* (n yes; %)	159; 48	177; 53	ns
WPI11 *lower leg, left* (n yes; %)	192; 58	181; 54	ns
WPI12 *hip (buttock, trochanter) right* (n yes; %)	220; 66	169; 51	< 0.001
WPI13 *upper leg, right* (n yes; %)	171; 51	158; 47	ns
WPI14 *lower leg, right* (n yes; %)	196; 59	164; 49	0.02
WPI15 *neck* (n yes; %)	271; 81	241; 72	0.01
WPI16 *upper back* (n yes; %)	234; 70	232; 70	ns
WPI17 *lower back* (n yes; %)	267; 80	226; 68	< 0.001
WPI18 *chest* (n yes; %)	129; 38	162; 48	0.02
WPI19 *abdomen* (n yes; %)	254; 76	160; 48	< 0.001
SSS[b] 1 *fatigue* (median; IQR)	3; 2-3	2; 1-3	< 0.001
SSS2 *waking unrefreshed* (median; IQR)	2; 2-3	2; 1-3	< 0.001
SSS3 *cognitive symptoms* (median; IQR)	3; 2-3	2; 1-3	< 0.001
SSS4 *headaches* (n yes; %)	211; 63	196; 59	ns
SSS5 *pain or cramps in lower abdomen* (n yes; %)	213; 64	181; 54	0.02
SSS6 *depression* (n yes; %)	251; 75	223; 67	0.02

**adjusted for Benjamini-Hochberg correction*

[a]Widespread Pain Index (WPI); [b]Symptoms Severity Scale (SSS).

Supplementary Table S2. FIQR single item scores among females and males affected by FM

	Female (median; IQR)	Male (median; IQR)	*p-value**
FIQR[a] 1 *brush or comb your hair*	4; 0-7	1; 0-5	< 0.001
FIQR2 *walk continuously for 20 minutes*	6; 4-9	5; 2-8	0.007
FIQR3 *prepare a homemade meal*	5; 1-7	3; 1-7	0.04
FIQR4 *vacuum, scrub, or sweep floors*	7; 5-10	5; 2-8	< 0.001
FIQR5 *lift and carry a bag full of groceries*	8; 6-10	7; 3,5-8	< 0.001
FIQR6 *climb one flight of stairs*	6; 3-8	5; 1-8	< 0.001
FIQR7 *change bed sheets*	6; 3-8	5; 1-8	< 0.001
FIQR8 *sit in a chair for 45 minutes*	7; 4-9	6; 3-8	0.03
FIQR9 *go shopping for groceries*	6; 3-8	5; 2-8	0.005
FIQR10 *daily life impact*	8; 6-9	7; 5-9	ns
FIQR11 *symptoms influence*	8; 7-10	8; 5-9	0.004
FIQR12 *pain level*	8; 5,5-9	8; 5-9	< 0.001
FIQR13 *energy level*	9; 7-10	7; 5-9	< 0.001
FIQR14 *stiffness level*	5; 2-8	5; 2-8	ns
FIQR15 *sleep quality*	6; 4-8	6; 2-7,5	< 0.001
FIQR16 *depression rate*	7; 4-9	6; 3-8	ns
FIQR17 *memory problems*	8; 5-9	7; 4-8	0.009
FIQR18 *anxiety level*	6; 2-8	5; 2-8	0.002
FIQR19 *tenderness to touch level*	7; 4,5-9	6; 3-8	0.02
FIQR20 *balance problems*	6; 3-8	5; 3-8	ns
FIQR21 *sensitivity to loud noises, bright lights, odors, cold*	7; 3,5-9	6; 3-8	0.005

**adjusted for Benjamini-Hochberg correction*

[a]Revised Fibromyalgia Impact Questionnaire (FIQR).

Supplementary Table S3. Tests for gender-related differential item functioning in the SSS subscale, and in the FIQR physical function and symptom domains among patients with FM

	SSS					
	Uniform			Non uniform		
Item	χ^2	p-value§	R^2 diff	χ^2	p-value§	R^2 diff
SSS[a] 1	10.2	0.001	0.0120	0.16	0.68	0.0001
SSS2	0.00	0.98	0.0000	1.70	0.19	0.0016
SSS3	0.04	0.82	0.0000	0.13	0.71	0.0001
SSS4	1.80	0.17	0.0023	26.2	< 0.001	0.0335
SSS5	0.64	0.42	0.0077	5.48	0.01	0.0009
SSS6	0.78	0.37	0.0012	0.45	0.49	0.0007

§Threshold for significance level with Bonferroni correction: 0.008

	FIQR physical function domain score					
	Uniform			Non uniform		
Item	χ^2	p-value§	R^2 diff	χ^2	p-value§	R^2 diff
FIQR[b] 1	0.00	0.93	0.0000	0.47	0.36	0.0001
FIQR2	2.12	0.14	0.0008	1.33	0.24	0.0005
FIQR3	14.0	< 0.001	0.0060	1.66	0.19	0.0007
FIQR4	25.7	< 0.001	0.0112	4.34	0.03	0.0001
FIQR5	27.1	< 0.001	0.0122	0.10	0.74	0.0000
FIQR6	0.86	0.35	0.0003	0.89	0.34	0.0003
FIQR7	0.05	0.80	0.0000	0.16	0.68	0.0000
FIQR8	2.39	0.12	0.0009	4.93	0.02	0.0019
FIQR9	11.9	< 0.001	0.0051	1.79	0.18	0.0007

§Threshold for significance level with Bonferroni correction: 0.005

	FIQR symptoms domain score					
	Uniform			Non uniform		
Item	χ^2	p-value§	R^2 diff	χ^2	p-value§	R^2 diff
FIQR12	2.95	0.08	0.0012	3.97	0.04	0.0017
FIQR13	9.35	0.002	0.0043	2.76	0.09	0.0012
FIQR14	0.29	0.58	0.0001	0.71	0.39	0.0003
FIQR15	15.1	< 0.001	0.0063	1.74	0.18	0.0007
FIQR16	7.68	0.005	0.0028	16.3	< 0.001	0.0060
FIQR17	0.12	0.72	0.0000	7.26	0.007	0.0012
FIQR18	1.48	0.22	0.0005	1.88	0.17	0.0006
FIQR19	0.14	0.70	0.0000	1.13	0.28	0.0002
FIQR20	4.13	0.04	0.0000	2.54	0.11	0.0004
FIQR21	0.00	0.97	0.0000	4.82	0.02	0.0018

§Threshold for significance level with Bonferroni correction: 0.005

[a]Symptoms Severity Scale (SSS); [b]Revised Fibromyalgia Impact Questionnaire (FIQR).
